# pH and Thermoresponsive PNIPAm-co-Polyacrylamide Hydrogel for Dual Stimuli-Responsive Controlled Drug Delivery

**DOI:** 10.3390/polym15010167

**Published:** 2022-12-29

**Authors:** Kokila Thirupathi, Thi Tuong Vy Phan, Madhappan Santhamoorthy, Vanaraj Ramkumar, Seong-Cheol Kim

**Affiliations:** 1 Department of Physics, Sri Moogambigai College of Arts and Science for Women, Palacode 636808, India; 2 Center for Advanced Chemistry, Institute of Research and Development, Duy Tan University, 03 Quang Trung, Hai Chau, Danang 550000, Vietnam; 3 Faculty of Environmental and Chemical Engineering, Duy Tan University, 03 Quang Trung, Hai Chau, Danang 550000, Vietnam; 4 School of Chemical Engineering, Yeungnam University, Gyeongsan 38541, Republic of Korea

**Keywords:** thermoresponsive copolymer, curcumin, pH-stimuli, cytocompatibility, drug delivery

## Abstract

The therapeutic delivery system with dual stimuli-responsiveness has attracted attention for drug delivery to target sites. In this study, we used free radical polymerization to develop a temperature and pH-responsive poly(N-isopropyl acrylamide)-co-poly(acrylamide) (PNIPAM-co-PAAm). PNIPAm-co-PAAm copolymer by reacting with N-isopropyl acrylamide (NIPAm) and acrylamide (Am) monomers. In addition, the synthesized melamine-glutaraldehyde (Mela-Glu) precursor was used as a cross-linker in the production of the melamine cross-linked PNIPAm-co-PAAm copolymer hydrogel (PNIPAm-co-PAAm-Mela HG) system. The temperature-responsive phase transition characteristics of the resulting PNIPAM-co-PAAm-Mela HG systems were determined. Furthermore, the pH-responsive drug release efficiency of curcumin was investigated under various pH and temperature circumstances. Under the combined pH and temperature stimuli (pH 5.0/45 °C), the PNIPAm-co-PAAm-Mela HG demonstrated substantial drug loading (74%), and nearly complete release of the loaded drug was accomplished in 8 h. Furthermore, the cytocompatibility of the PNIPAm-co-PAAm-Mela HG was evaluated on a human liver cancer cell line (HepG2), and the findings demonstrated that the prepared PNIPAm-co-PAAm-Mela HG is biocompatible. As a result, the PNIPAm-co-PAAm-Mela HG system might be used for both pH and temperature-stimuli-responsive drug delivery.

## 1. Introduction

The controlled delivery of therapeutic drugs has been considered an effective technique for maintaining therapeutic effectiveness [1,2,3]. Controlled drug delivery carriers enable therapeutic concentrations to be maintained while also protecting drugs from enzymatic degradation, improving drug solubility, decreasing adverse effects, and extending release time [4]. Although drug delivery carriers are advantageous, they do have significant clinical limits [5]. As a result, self-regulating drug delivery systems that deliver drugs based on changes in physiological parameters are required [6]. As drug carrier systems, a variety of drug delivery methods, such as nanoparticles, polymeric materials, and lipids, have been employed [7,8,9].

Hydrogels are cross-linked polymeric networks having the ability to expand in an aqueous medium or biological fluid and hold a considerable amount of water [10,11]. The hydrogels mimic adjacent tissues due to their biocompatibility and high-water content. Because of their swelling–deswelling capabilities, hydrogels are widely used in biotechnological and biomedical applications such as tissue engineering, regenerative medicine, and diagnostic biosensor fields [12,13]. Chemically cross-linked hydrogel polymers are currently attracting much research attention as prospective drug delivery matrices. The drug release behavior might be influenced by internal or external stimuli such as pH, temperature, light, and ultrasound [14,15,16,17,18,19]. Dual-stimuli materials, such as temperature and pH-sensitive hydrogel materials, are frequently used in biomedical fields because these parameters may be easily controlled in vitro and in vivo [20].

However, for practical applications, homogenous dispersion of therapeutic molecules in hydrogels is essential, which is one of the key disadvantages of present hydrogel materials. Because of the enhanced swelling capacity of hydrogels, the drug is released quickly. The selective and controlled release of a loaded drug is considered to be an efficient strategy for minimizing undesirable side effects on normal cells and tissues [21]. As a result, the development of dual-stimuli controlled delivery systems, such as pH and temperature-responsive systems, is thought to be important for regulated and selective drug delivery in target sites. Because of their ability to respond to temperature changes in the presence of biological fluid, thermosensitive hydrogels are gaining popularity in drug delivery and tissue encapsulation [22]. Among them, thermoresponsive poly(N-isopropyl acrylamide) (PNIPAm) is the most well-investigated polymer due to its distinctive phase transition from an extended hydrophilic state to a collapsed hydrophobic state in water at around 32 °C. The PNIPAm-based hydrogels undergo an abrupt phase transition both below and above LCST (about 32 °C). In the presence of temperature stimuli, the drug-loaded PNIPAm hydrogels undergo globular structural change, releasing the loaded drugs from the hydrogel network [23]. At swelling temperatures below LCST, the cross-linked hydrogel formed with PNIPAm polymer is hydrophilic. They, on the other hand, acquire a collapsed state whenever the temperature in the aqueous medium is above the LSCT.

Aside from thermoresponsive hydrogels, dual-stimuli responsive hydrogels are produced by copolymerizing two or more appropriate monomers. These pH-responsive hydrogels are a type of stimuli-responsive material used in biological applications. In response to pH variations, such hydrogel systems regulate drug release from the drug-loaded hydrogel. These pH-responsive hydrogels are often synthesized from polymers with weakly basic (-NH_2_) or weakly acidic (-COOH) functional units [16,24]. The drug release can be accomplished by the protonation and deprotonation of these functional groups present in hydrogels. Both pH and temperature-responsive hydrogel systems are required for specific biomedical applications such as synergistic chemo-photothermal therapy and magnetic hyperthermia-induced drug delivery applications. Copolymerization of NIPAm with appropriate pH-sensitive monomers bearing basic functional groups in the presence of a limited number of cross-linkers results in dual pH and temperature-stimuli-responsive hydrogels [25].

We developed a thermo- and pH-responsive PNIPAm-based copolymer by combining polyacrylamide (PAAm) units cross-linked with melamine (Mel) units to produce PNIPAm-co-PAAm-Mel copolymer hydrogel (HG). The thermoresponsive PNIPAm has used swelling–deswelling behavior under temperature changes to achieve its sharp phase transition. Because of its basic amine functional groups, PAAm has a pH-responsive segment that can act as a drug-binding site. Additionally, Mel units have been employed to increase the cross-linkage of the copolymer network as well as the number of drug-binding sites. In order to characterize the synthesized PNIPAm-co-PAAm-Mel HG material, various experimental methods such as ^1^H NMR, FTIR, SEM, and zeta potential were used. Curcumin (Cur) was used as a model cargo to assess in vitro drug loading and pH and temperature-responsive release characteristics. The in vitro drug release study performance at 8 h revealed that under combined pH and temperature stimuli, nearly complete release of Cur occurs at 45 °C and pH 5.0, compared to approximately 75% release only at pH stimuli (pH 5.0) or approximately 58% release only at temperature stimuli (45 °C), respectively. As a result, the prepared dual-stimuli responsive PNIPAm-co-PAAm-Mel HG system may be used for selective drug delivery to the target region.

## 2. Materials and Methods

### 2.1. Reagents and Chemicals

Acrylamide (AAm, 99%), melamine (Mel, 99%), 2,2-azobisisobutyronitrile (AIBN, 12 wt.% in acetone), N-Isopropylacrylamide (NIPAm, 97%), ethanol (99%), tetrahydrofuran (THF, 99.9%), diethyl ether (99.7%) and curcumin (98%), were purchased from Sigma Aldrich Chemical Co., Saint Louis, MO, USA, and used as received.

### 2.2. Synthesis of PNIPAm-co-PAAm Copolymer

**Scheme 1:**
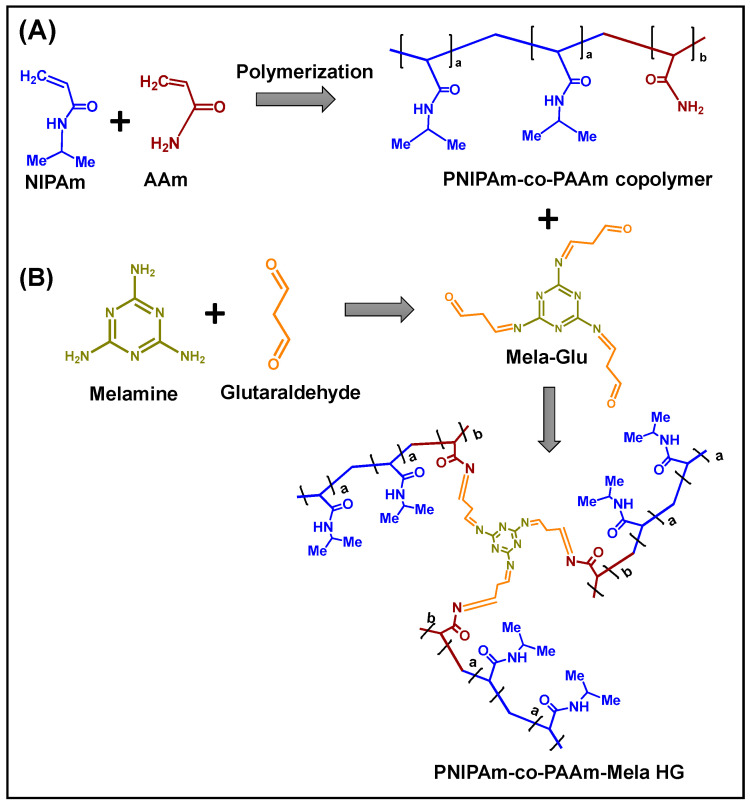
The scheme represents the (**A**) preparation of PNIPAm-co-PAAm copolymer; (**B**) Mela-Glu cross-linked PNIPAm-co-PAAm HG system.

The PNIPAm-co-PAAm copolymer was synthesized via a free radical polymerization process using AIBN as the initiator [26]. 2.0 g (17.5 mmol) NIPAm and 1.38 g (17.7 mmol) AAm were solubilized in a 50 mL two-necked round bottom flask containing 15 mL dry THF solvent for this experiment. The reaction flask was then continuously purged with nitrogen gas for 45 min before adding around 0.05 g of AIBN in THF (0.5 mL) and performing the reaction at 68 °C for 24 h under inert conditions. The resulting viscous mass was then precipitated in hexane (100 mL). This precipitation procedure was repeated five times to remove the unreacted monomer, and the white precipitate that formed was vacuum dried at room temperature. The copolymer was named PNIPAm-co-PAAm copolymer (Scheme 1A).

### 2.3. Synthesis of Glutaraldehyde-Modified Melamine Precursor

Approximately 1.0 g (7.9 mmol) melamine was mixed in 25 mL of water: ethanol (40:60 vol/vol) mixture for this reaction. In the presence of an acetic acid catalyst, 2.38 mL (2.3 mmol) of glutaraldehyde was added. The reaction was carried out at 90 °C for 48 h. The product was purified and recrystallized from hot methanol after the reaction mixture was concentrated using a rotary evaporator. The final product was designated as Mela-Glu precursor (Scheme 1B).

### 2.4. PNIPAm-co-PAAm Copolymer Cross-Linked with Mela-Glu Precursor

The PNIPAm-co-PAAm copolymer was cross-linked with Mela-Glu precursor as follows. Approximately 1.0 g of PNIPAm-co-PAAm copolymer was dissolved in 25 mL ethanol. Next, 0.3 g of Mela-Glu precursor in ethanol (5 mL) was slowly introduced, allowing for a cross-linking reaction between the amine groups of PAAm segments and the aldehyde groups of Mela-Glu units through Schiff base reaction in the presence of an acetic acid catalyst [27]. The obtained hydrogel sample was placed in a dialysis membrane tubing with molecular weight cutoff (MWCO) at 3.5 kDa and placed in a beaker containing 200 mL of ethanol-water (1:1 *v*/*v*) mixture. The surrounding solvent was exchanged every 6 h, and a fresh 200 mL of ethanol-water was used. Finally, the purified sample was separated and dried at room temperature under vacuum for 12 h. The Mela-Glu precursor cross-linked hydrogel obtained was designated as PNIPAm-co-PAAm-Mela HG (Scheme 1).

### 2.5. Characterization 

^1^H-NMR analysis of the PNIPAm-co-PAAm copolymer, Mela-Glu precursor, and PNIPAm-co-PAAm-Mela HG sample was carried out using the OXFORD instrument (600 MHz). Scanning electron microscopy (SEM, JEOL 6400 instrument) at 10 kV was applied to measure the surface structure of the prepared copolymer sample. X-ray photoelectron spectroscopy (XPS) analysis was performed on the XPS instrument (Tucson, AZ, USA 85706). Fourier-transform infrared (FTIR) analysis was carried out using JASCO FTIR 4100 instrument. Particle size and the zeta potential measurements were performed on the Malvern Zetasizer Nano-ZS. UV-vis spectral analysis was performed using an Agilent Inc. UV-Vis spectrophotometer.

### 2.6. Turbidity Measurement

For this experiment, about 25 mg/mL of the PNIPAm-co-PAAm-Mela HG sample was dissolved in 5 mL distilled water to measure the turbidity of the synthesized PNIPAm-co-PAAm-Mela HG sample. The absorbance of the obtained polymer solution was measured at various temperatures ranging from 25 to 80 °C. The absorbance of the sample at each temperature was measured.

### 2.7. Drug Loading into PNIPAm-co-PAAm-Mela HG

**Scheme 2:**
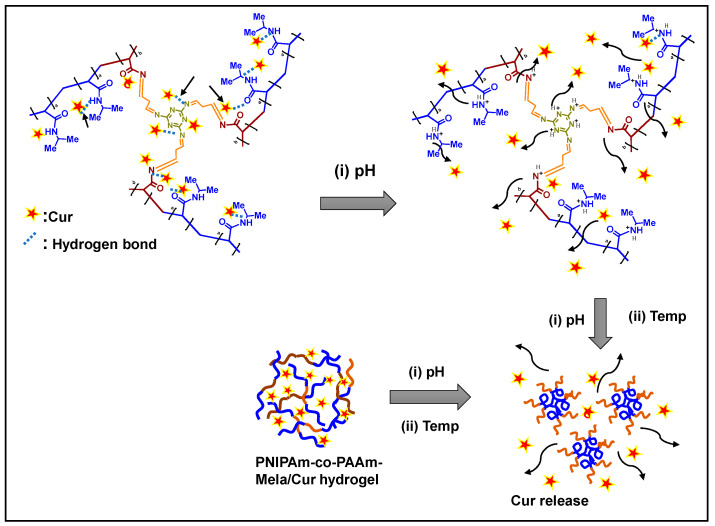
A proposed schematic representation of drug loading into the PNIPAm-co-PAAm-Mela HG system. The pH stimuli and the combined pH and temperature-stimuli-responsive release behavior of the PNIPAm-co-PAAm-Mela HG system.

A model drug, Cur, was used for drug loading and release experiments in vitro. The swelling diffusion method was used to load Cur into the PNIPAm-co-PAAm-Mela HG system at a polymer: drug *w*/*w* ratio of (10:3) [28]. In 3 mL of water, 0.1 g of PNIPAm-co-PAAm-Mela HG sample was dissolved, and 30 mg of Cur drug was combined. For 24 h, the suspension mixture was stirred at room temperature. The drug loading content into the PNIPAm-co-PAAm-Mela HG was determined using a UV-Vis spectrophotometer at 425 nm after the Cur encapsulated copolymer was separated by centrifugation at 50 °C. The drug-loaded sample was labeled as PNIPAm-co-PAAm-Mela@Cur HG (Scheme 2). The drug loading percentage was calculated to be around 78% using the following equation.

Drug loading (%) = (Wt. of the drug in sample/Wt. of the sample) × 100(1)

### 2.8. pH and Temperature-Responsive Drug Release Experiments

The PNIPAm-co-PAAm-Mela/Cur HG was evaluated in vitro under various circumstances, including (i) varied pH (pH 7.4 and 5.0); (ii) different temperatures (25 °C and 45 °C); and (iii) the combination of pH + temperature (pH 7.4/45 °C and pH 5/45 °C, respectively). For these experiments, approximately 25 mg/mL of drug-loaded sample PNIPAm-co-PAAm-Mela@Cur HG was placed in a dialysis bag (Mol. wt. cut off 5000 kDa), and the bag was immersed in a beaker containing 25 mL of phosphate-buffered saline (PBS) solution at different pH and temperature under gentle magnetic stirring. At the set time, about 1 mL of release media was removed, and the released Cur was measured using a UV-Vis spectrophotometer at 425 nm. The calibration curve plot was used to calculate the released Cur. The cumulative drug release was calculated using the equation below. Drug release (%) = (Amount of Cur released at time t/Total amount of Cur in the HG sample) × 100.

### 2.9. Cytocompatibility Study

The synthesized PNIPAm-co-PAAm-Mela HG, Cur loaded PNIPAm-co-PAAm-Mela@Cur HG, and pure Cur samples were tested for in vitro cytocompatibility utilizing the 3-(4,5-dimethylthiazol-2-yl)-2,5-diphenyl tetrazolium bromide (MTT) assay. HepG2 cells (2 × 10^4^ cells/well) were grown in a 96-well plate for 24 h at 37 °C for this experiment. The existing medium was replaced with new media containing varying concentrations of PNIPAm-co-PAAm-Mela HG, PNIPAm-co-PAAm-Mela@Cur HG, and pure Cur. After 4 h, the MTT solution was added to each well and incubated for another 4 h. Following that, about 20 μL of DMSO was added to dissolve the existing formazan crystals, and the absorbance at 592 nm was measured using a microplate reader.

### 2.10. Statistical Analysis

All results, expressed as the mean ± SD, were analyzed using a two-tailed Student’s t-test or one-way analysis of variance (ANOVA). The acceptable level of significance was *p* < 0.05.

## 3. Results and Discussion

### 3.1. Structural Study of PNIPAm-co-PAAm-Mela HG System

**Figure 1:**
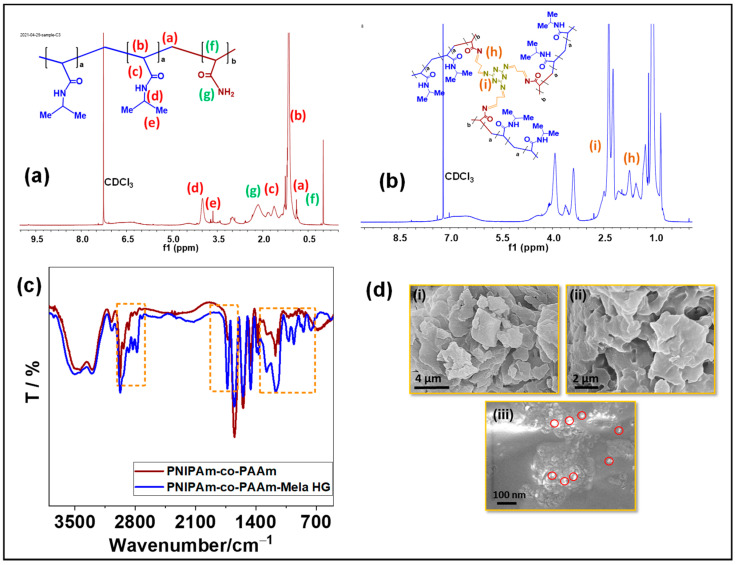
Characterization of PNIPAm-co-PAAm copolymer samples. (**a**,**b**) ¹H NMR spectrum; and (**c**) FT-IR analysis of PNIPAm-co-PAAm copolymer; and PNIPAm-co-PAAm-Mela HG samples, respectively. (**d**) SEM images of the (**i**) PNIPAm-co-PAAm copolymer; (**ii**) PNIPAm-co-PAAm-Mela HG; (**iii**) PNIPAm-co-PAAm-Mela/Cur HG samples, respectively. The red circles in figure (**d**) (**iii**) indicate the presence of Cur molecules in the hydrogel samples.

The ^1^H NMR spectrum analysis confirmed the structure of the prepared PNIPAm-co-PAAm-Mela HG. The ^1^H NMR spectra of the hydrogel system in CDCl_3_ solvent are shown in Figure 1. Because of the solubility of hydrophilic PAAm and hydrophobic PNIPAm and Mela segments, all of the relevant proton signals for PNIPAm-co-PAAm-Mela HG block copolymer are presented in Figure 1a. The resonance peaks for methyl (-CH_2_) and -OCH_2_- groups were δ0.7 ppm, δ1.2 ppm and δ1.8 ppm, respectively. The significant resonance signal at δ3.2 ppm indicates that the copolymer included the PNIPAm polymer segment in the PNIPAm-co-PAAm copolymer HG. The resonance peaks of methyl protons in isopropyl units are indicated by the peak at δ3.6 ppm. Furthermore, after Mela groups cross-linking, the resonance signals at δ2.4 ppm and δ1.82 ppm indicated that the melamine groups were cross-linked via glutaraldehyde units Figure 1b.

The structure of the PNIPAm-co-PAAm-Mela HG system was determined using FT-IR spectroscopy. The FTIR spectrum is shown in Figure 1c, with the characteristic vibration peak at 1542 cm^−1^ corresponding to the imine group (-C=N-) vibration, the peak at 1453 cm^−1^ indicating the stretching bands of C-N groups, and the sharp band at 1417 cm^−1^ indicating the N-H groups of NIPAm units in the PNIPAm-co-PAAm copolymer sample. The stretching mode of C=O groups in PNIPAm and PAAm segments was attributed to the absorption peak at 1727 cm^−1^. Additionally, the intense stretching peak appeared at 2836 cm^−1^ and 2912 cm^−1^ of the PNIPAM-co-PAAM-Mela copolymer system’s alkyl carbon C-C stretching modes.

SEM analysis was used to examine the surface morphology of the prepared copolymer samples. SEM analysis was carried out on dried powder samples of PNIPAm-co-PAAm copolymer, PNIPAm-co-PAAm-Mela HG, and Cur loaded PNIPAm-co-PAAm-Mela/Cur HG, respectively. The PNIPAm-co-PAAm copolymer exhibited flake-like particles with an average particle size of around 2–3 μm, as shown in Figure 1d(i). In contrast, the Mela-Glu precursor cross-linked PNIPAm-co-PAAm HG sample (Figure 1d(ii)) exhibited slight aggregation with interconnected particles with appropriate pores with an average diameter of about 1 μm, which could be attributed to the cross-linking of PNIPAm-co-PAAm polymer chains via Mela-Glu precursor. Furthermore, the Cur drug-loaded PNIPAm-co-PAAm-Mela/Cur HG sample exhibited a similar aggregated morphology with micropores (Figure 1d(iii)).

**Figure 2:**
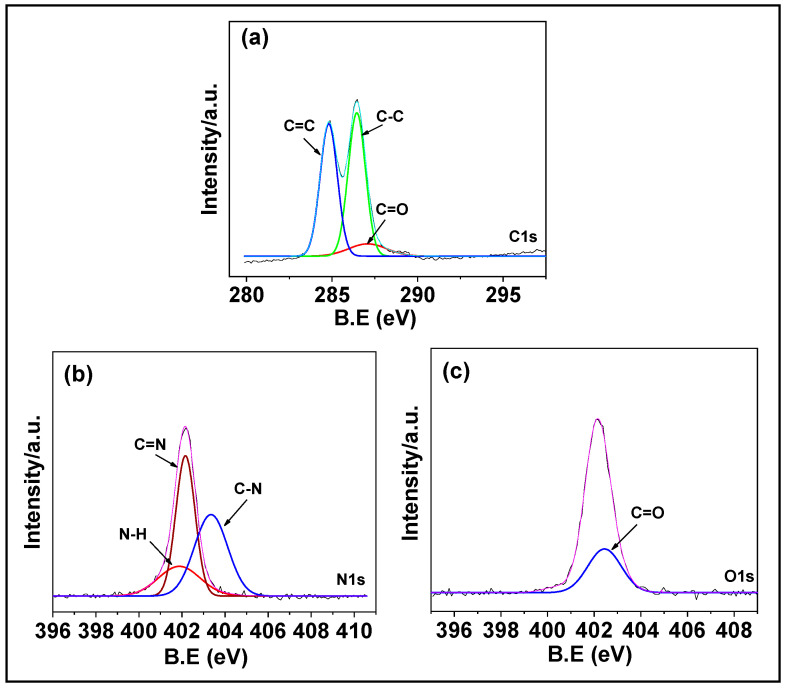
XPS analysis of PNIPAm-co-PAAm-Mela HG. The core level spectra of (**a**) C 1s; (**b**) N 1s; and (**c**) O 1s, respectively.

The composition of PNIPAm-co-PAAm-Mela HG was determined by XPS analysis. As seen in Figure 2, the XPS spectra with prominent signals for carbon (C1s), nitrogen (N1s), and oxygen (O1s) peaks suggested that the presence of N-isopropyl acrylamide and acrylamide monomer segments existed in the PNIPAm-co-PAAm-Mela HG system. The high-resolution C1s spectra of PNIPAm-co-PAAm-Mela HG were shown in Figure 2a, with the peak at 284.5 eV and −289.2 mV, representing the aliphatic C-C binding mode. The nitrogen peak at 403.3 eV indicates the C-N groups of N-isopropyl acrylamide and acrylamide monomers in the PNIPAm-co-PAAm-Mela HG. The N1s spectra in Figure 2b revealed two peaks at 398.4 eV and 399.25 eV, evidenced that the C-N and C=N bonds of the amide and imine groups of N-isopropyl acrylamide and acrylamide monomer, as well as the melamine units of PNIPAm-co-PAAm-Mela HG [29]. Furthermore, the O1s signal at 402.9 eV represents the C=O groups of N-isopropyl acrylamide and acrylamide monomer in the PNIPAm-co-PAAm-Mela HG (Figure 2c). This supports that the PNIPAm-co-PAAm-Mela copolymer material was successfully synthesized [30].

### 3.2. Physicochemical Properties of PNIPAm-co-PAAm-Mela HG System

**Figure 3:**
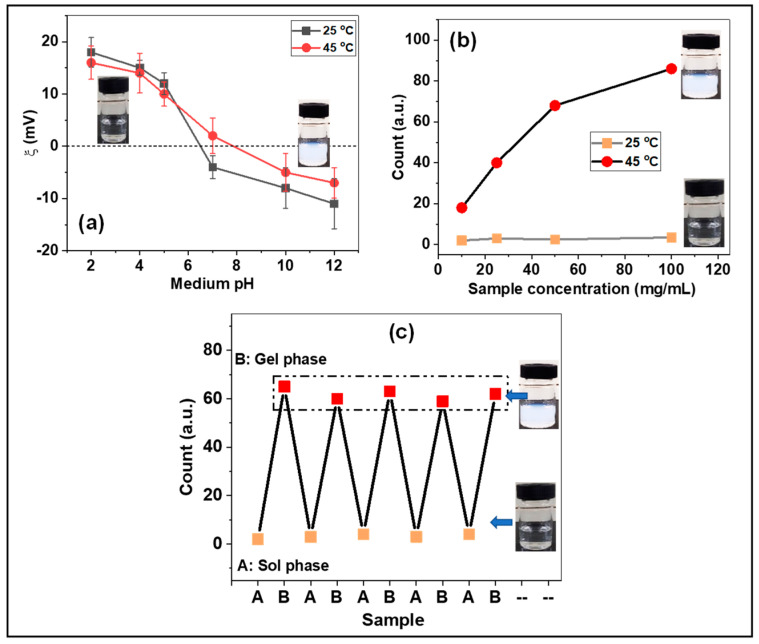
(**a**) Zeta potential and (**b**) particle size of PNIPAm−co−PAAm-Mela HG sample. Mean with error bar *n* = 3. (**c**) DLS analysis of PNIPAm−co−PAAm-Mela HG at below and above LCST.

The surface charge of the synthesized PNIPAm-co-PAAm-Mela HG was evaluated using zeta potential analysis. The presence of amide (-N-H), imine (-C=N-), and carbonyl (-C=O) groups in the copolymer segments increased the pH sensitivity of the PNIPAm-co-PAAm-Mela HG. The zeta potential of the PNIPAm-co-PAAm-Mela HG samples was measured at 25 °C and 45 °C, respectively. As shown in Figure 3a, the zeta potential value decreased from +19 mV to −13 mV and from +16 mV to −9 mV for samples evaluated at 25 °C and 45 °C, respectively, as the pH increased from 2 to 10. At low pH and higher temperature (45 °C), the hydrophobic PNIPAm segments combine to form the hydrophobic micelle core, whereas the hydrophilic PAAm forms the outer shell structure. The PNIPAm-co-PAAm-Mela HG, on the other hand, enhances hydrophilicity at higher pH, allowing them to transit the sol phase. The zeta potential values are slightly lower at higher temperatures (45 °C) than at lower temperatures (25 °C), which may be attributed to the development of aggregated micelles above LCST. The dynamic light scattering (DLS) technique was used to determine the linear to globule phase transition at different temperatures, such as 25 °C and 45 °C. As seen in Figure 3b, the DLS intensity remained consistent at 25 °C for the sample concentration of 25 mg/mL. Moreover, the solution temperature above LCST, the PNIPAm-co-PAAm-Mela HG showed increased turbidity (gel phase) and showed appropriate particle size when the solution temperature increased from 25 °C to 45 °C. On the other hand, when cooling the sample to about 25 °C, the sample did not show any considerable particle size and appeared to be a clear transparent solution. The DLS measurement under repeated heating and cooling conditions was examined at 45 °C and 25 °C, respectively (Figure 3b). DLS methods were also used to investigate the temperature responsiveness of the PNIPAm-co-PAAm-Mela HG polymer. Figure 3c shows that at 25 °C, no significant particles are formed. However, tiny particles are repeatedly formed at 45 °C. This work demonstrated that the PNIPAm-co-PAAm-Mela HG polymer undergoes a significant phase transition at temperatures above the LCST.

### 3.3. Phase Transition Mechanism of PNIPAm-co-PAAm-Mela HG System

**Figure 4:**
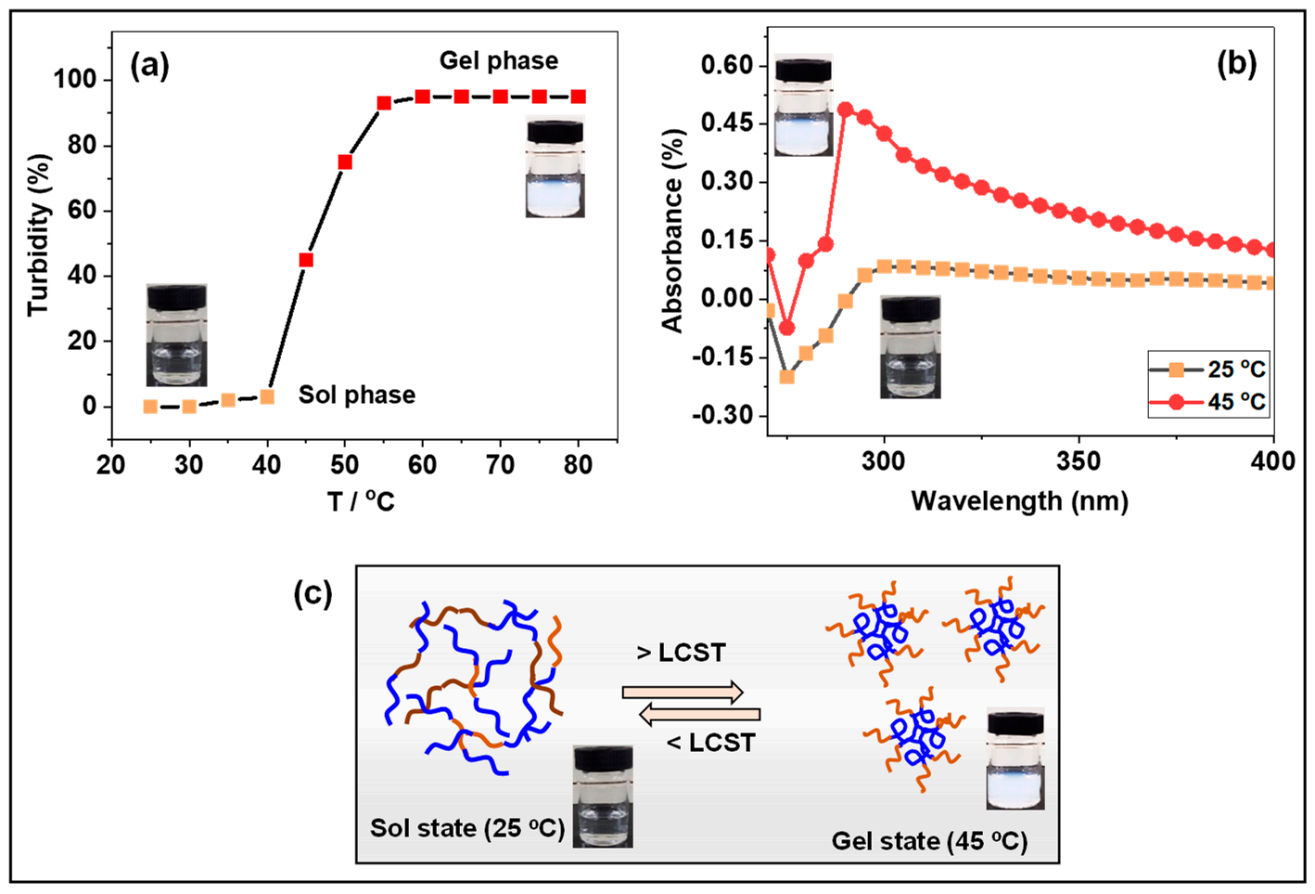
(**a**) Relative turbidity of PNIPAm−co−PAAm-Mela HG at 25 °C to 80 °C. (**b**) UV-vis absorption of PNIPAm-co-PAAm-Mela HG sample at 25°C and 45°C. (**c**) Illustrates the phase transition of PNIPAm−co−PAAm−Mela HG below and above LCST. The violet color indicates the hydrophobic PNIPAm domain, and the brown color indicates the hydrophilic PAAm segments in the presence of water.

The relative turbidity of PNIPAm-co-PAAm-Mela HG polymer (25 mg/mL) was investigated from 25 to 80 °C (Figure 4a). At a medium temperature of less than 40 °C, the sample displayed a transparent and homogenous clear solution, as illustrated in the graph (Figure 4a) and the sample vial. The PNIPAm-co-PAAm-Mela HG polymer absorbs water and becomes hydrated, swelling at this low LCST (linear structure). At temperatures above 40 °C, however, the clear solution became turbid, and the relative turbidity in the solution medium increased with increasing temperature to above 45 °C, indicating that the linear copolymer chain collapses into a hydrophobic globule micelle structure [31]. UV-Vis absorption and relative turbidity were used to study the swelling and deswelling properties of the prepared PNIPAm-co-PAAm-Mela HG at various solution temperatures. At 25 °C and 45 °C, UV-Vis absorbance of the PNIPAm-co-PAAm-Mela HG sample (25 mg/mL) was determined. As shown in Figure 4b, a significant weak absorption peak was seen at 25 °C, which might be attributed to the linear polymer structure and clear solution medium. At 45 °C, however, the solution medium becomes turbid due to temperature-induced micelle formation caused by the transition of the PNIPAm-co-PAAm-Mela HG’s hydrophilic linear to hydrophobic globule structure above LCST (Figure 4b).

The unique properties of dual-stimuli-responsive hydrogels include the ability to undergo noticeable phase transitions in response to physical stimuli rather than chemical or mechanical stimuli. At 25 °C, the PNIPAm-co-PAAm-Mela HG polymer dissolves readily in water and forms a non-cross-linked homogenous solution. The copolymer segments of PNIPAm-co-PAAm-Mela HG comprise hydrophilic amide (-CO-NH-) groups and hydrophobic isopropyl (-CH(CH_3_)_2_) groups. In deionized water, the PNIPAm-co-PAAm-Mela HG polymer undergoes a sharp phase transition and exists in solution as a linear hydrophilic polymer chain below LCST. In contrast, above LCST, the PNIPAm-co-PAAm-Mela HG transformed into a hydrophobic globule coil shape (Figure 4c).

### 3.4. Cur Loading and pH-Responsive Delivery from PNIPAm-co-PAAm-Mela/Cur HG System 

Because the PNIPAm-co-PAAm-Mela HG has a higher LCST than human body temperature, fast micelle formation may be prevented when it is injected into the body. By fine-tuning the temperature stimuli, it is possible to maintain selective and controlled drug release to the target sites. Cur, an anticancer drug, may be encapsulated into the PNIPAm-co-PAAm-Mela HG by mixing them with a polymer solution at low temperatures. At low temperatures, the loaded Cur in the PNIPAm-co-PAAm-Mela HG can be protected against denaturation. The drug molecules can be interacted with the PNIPAm-co-PAAm-Mela HG by hydrogen bonding or electrostatic interactions (Scheme 2). The amide, imine, and carbonyl functional groups in the PNIPAm-co-PAAm-Mela HG system act as drug binding sites as well as protonation centers, ionize in low pH conditions and promote the release of loaded drugs from the PNIPAm-co-PAAm-Mela HG system (Scheme 2).

The pH and temperature-responsive drug release behavior of the prepared Cur loaded PNIPAm-co-PAAm-Mela/Cur HG has been evaluated at different pH and temperature conditions, specifically at different pH (pH 7.4 and 5.0); at different temperatures (25 °C and 45 °C); and the combined pH and temperature (pH 7.4/45 °C, pH 7.4/45 °C, respectively). First, the pH-responsive Cur release behavior from the PNIPAm-co-PAAm-Mela/Cur HG was studied.

**Figure 5:**
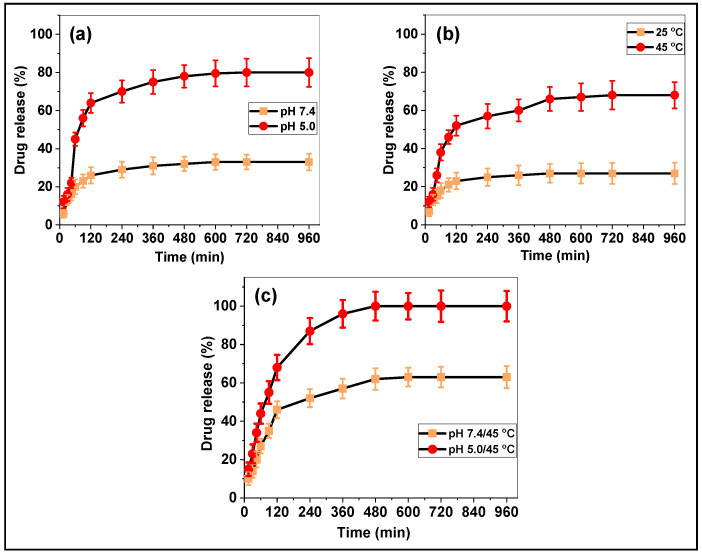
In vitro Cur delivery of PNIPAm-co-PAAm-Mela/Cur HG system. (**a**) Cur release at different pH, (**b**) Cur release at different temperature stimuli, and (**c**) Cur release with the combined pH and temperature conditions, respectively. Mean with error bar *n* = 3.

Figure 5a demonstrated the Cur release behavior at various pHs, with approximately ~30% and ~82% of Cur released in 12 h at pH 7.4 and 5.0, respectively. The enhanced Cur release observed at pH 5.0 might be attributed to acid-induced protonation of the nitrogen part of PNIPAm and cross-linked Mela groups, as well as the loaded Cur molecules (Figure 5a). Second, Figure 5b depicted the temperature-responsive release behavior, which revealed that around ~26% and ~68% of Cur release was observed at 25 °C and 45 °C, respectively, throughout a 12 h release period. The release of physisorbed Cur molecules was responsible for the increase in release at 45 °C (Figure 5b). Third, at pH 7.4/45 °C and pH 5.0/45 °C, respectively, the combined pH and temperature-stimuli-responsive Cur release were determined. As seen in Figure 5c, a gradual release of Cur was detected, with about ~65% released at pH 7.4/45 °C; an almost complete release of Cur was observed at pH 5.0/45 °C, respectively, in a 12 h release period. Under the combined pH and temperature conditions, enhanced Cur release was observed due to the temperature-induced phase transition and pH-induced protonation of the functional groups and Cur molecules, which induce an electrostatic repulsive force. Therefore, the PNIPAm-co-PAAm-Mel HG system showed considerably enhanced Cur release under the combined pH and temperature stimuli conditions.

The drug loading mechanisms could be described as follows. As shown in Scheme 2, the loaded Cur molecules are strongly associated with the amine, imine, and amide groups via H-bonding/electrostatic interactions at pH 7.4. As a result, only a negligible amount of Cur was released from the PNIPAm-co-PAAm-Mela/Cur HG system at pH 7.4. The enhanced Cur release observed at the combined acidic pH and temperature (pH 5.0/45 °C) might be attributed to the temperature-induced phase change and acid-induced protonation of drug-binding functional sites and drug molecules, both of which force out the Cur molecules from the PNIPAm-co-PAAm-Mela/Cur HG system (Scheme 2).

The experiment results showed that combining pH and temperature stimuli resulted in greater Cur release efficiency from the PNIPAm-co-PAAm-Mela/Cur HG system than single stimuli, such as only pH or temperature stimuli (Table 1 and Table 2). The majority of thermoresponsive polymers reported in the literature [23,32,33,34] primarily focused on temperature (Table 3), but in this work, our proposed melamine cross-linked PNIPAm-co-PAAm-Mela/Cur HG system has advantages such as enhanced drug loading and dual-stimuli-responsive drug release to the target sites.

### 3.5. Cytocompatibility

**Figure 6:**
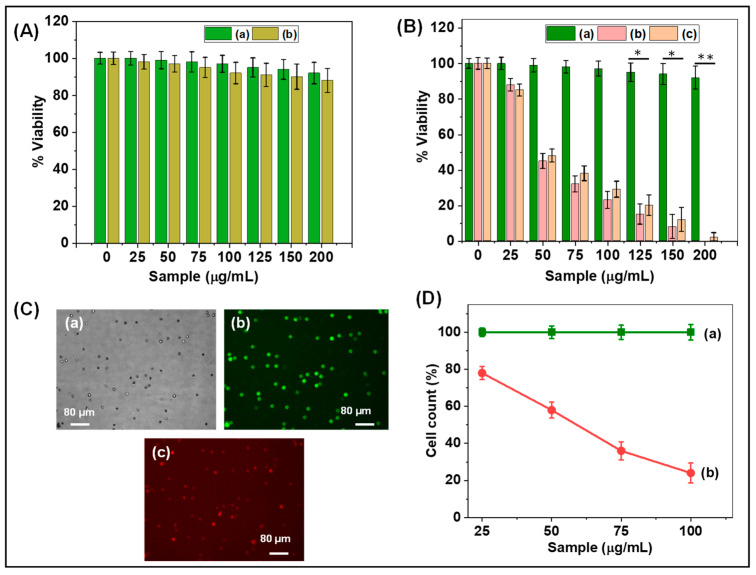
(**A**) In vitro cytocompatibility of (**a**) control HepG2 cells and (**b**) PNIPAm-co-PAAm copolymer; (**B**) In vitro cytocompatibility of (**a**)PNIPAm-co-PAAm-Mela HG; (**b**) PNIPAm-co-PAAm-Mela/Cur HG; and (**c**) pure Cur drug, respectively, at different concentrations. Statistical significance to the cell toxicity with different samples (*, significant *p* < 0.05; **, highly significant *p* < 0.01). (**C**) Fluorescence microscopy images of HepG2 cells represent (**a**) dark-field image; (**b**) PNIPAm-co-PAAm-Mela HG treated cells; and (**c**) Cur loaded PNIPAm-co-PAAm-Mela/Cur HG system, respectively. (**D**) Cell viability of (**a**) PNIPAm-co-PAAm-Mela HG treated cells; and (**b**) Cur loaded PNIPAm-co-PAAm-Mela/Cur HG system, respectively, at different sample concentrations. Mean with error bar *n* = 3.

The cytocompatibility of the prepared PNIPAm-co-PAAm copolymer, PNIPAm-co-PAAm-Mela HG, Cur-loaded PNIPAm-co-PAAm-Mela/Cur HG system, and pure Cur was tested in vitro at 37 °C using the HepG2 cell line. The cytocompatibility of control HepG2 cells and different concentrations of synthesized PNIPAm-co-PAAm copolymer samples are shown in Figure 6A. As shown in Figure 6A, the synthesized PNIPAm-co-PAAm copolymer exhibits about ~90% cell viability even at a sample concentration of 200 μg/mL, indicating that the PNIPAm-co-PAAm copolymer is biocompatible in nature [35,36]. On the other hand, Figure 6B shows that the PNIPAm-co-PAAm-Mela HG system without Cur loading demonstrated ~90% cell survival in all investigated sample concentrations, demonstrating that the prepared PNIPAm-co-PAAm-Mela HG also shows excellent biocompatibility to the HepG2 cells. In contrast, the Cur-loaded PNIPAm-co-PAAm-Mela/Cur HG system was shown to be toxic to HepG2 cells at all sample concentrations. It was observed that cells treated with a sample concentration of 200 μg/mL demonstrated nearly complete cell killing efficiency, implying that a concentration of 200 μg/mL of PNIPAm-co-PAAm-Mela HG is sufficient for complete cell killing [37]. This in vitro study indicates that the prepared PNIPAm-co-PAAm-Mela HG might be used for drug loading and pH stimuli-responsive drug release to specific sites.

### 3.6. Fluorescence Image Analysis

The cell-killing efficacy of the released Cur was determined by staining HepG2 cells with propidium iodide and acridine orange to distinguish between living and dead cells. As seen in Figure 6C, the cells treated with PNIPAm-co-PAAm-Mela HG fluoresced green, indicating that the majority of the cells were alive. The Cur-loaded PNIPAm-co-PAAm-Mela/Cur HG sample treated cells, on the other hand, exhibit red fluorescence, indicating that around >95% of cells were killed [38,39]. This might be due to the released Cur drugs from the PNIPAm-co-PAAm-Mela/Cur HG system, which induced cell death. As seen in Figure 6D, the cell killing efficiency is a function of sample concentration. The cell killing efficiency rose when the PNIPAm-co-PAAm-Mela/Cur HG sample concentration increased, which may be attributed to an increase in released Cur from the PNIPAm-co-PAAm-Mela/Cur HG system, and the released Cur induced more cell death.

## 4. Conclusions

In this study, we developed a dual-stimulus PNIPAm-co-PAAm-Mela/Cur HG copolymer system for temperature-responsive and pH-induced drug delivery applications. To evaluate the drug delivery behavior of the PNIPAm-co-PAAm-Mela HG copolymer, we employed Cur as a model drug. The PNIPAm-co-PAAm-Mela HG system had much higher Cur loading efficiency (73%) due to the existence of more drug-binding sites of amide, amine, and imine sites in the PNIPAm and Mel cross-linked PAAm segments. The drug release investigation revealed that a nearly complete release of Cur was accomplished in the presence of the combined pH and temperature stimulation conditions (pH 5.0/45 °C Furthermore, the results of the cytocompatibility investigation show that the prepared PNIPAm-co-PAAm-Mela HG system is cytocompatible and that the loaded drug may be released in the intracellular microenvironment. The overall study findings demonstrated that the PNIPAm-co-PAAm-Mela HG system might be used for controlled drug release to specific sites in chemotherapeutic applications.

## Figures and Tables

**Table 1:** Cur release efficiency from PNIPAm-co-PAAm-Mela HG system at different temperatures.

Sample	LCST (°C)	Release Efficiency (%)	
PNIPAm-co-PAAm- Mela HG/Cur	40	25 °C	45 °C
		26	68

**Table 2:** Cur release efficiency from PNIPAm-co-PAAm-Mel HG system at pH/temperatures.

pH	Release Efficiency (%)
7.4	30
5.0	82
7.4/45 °C	65
5.0/45 °C	100

**Table 3:** Various PNIPAM-based copolymer hydrogels for Cur delivery.

Polymers	Stimuli	Refs.
Quaternized triblock terpolymers	Temperature	[32]
pNIPAM grafted chitosan hydrogels	Temperature	[33]
Peptide-PNIPAM hydrogel	Temperature	[23]
Rechargeable pNIPAM hydrogel	Temperature	[34]
PNIPAm-co-PAAm-Mela HG	pH and temperature	This work

## Data Availability

The data presented in this study are available on request from the corresponding author.
